# Predictive Value of Combined LIPS and ANG-2 Level in Critically Ill Patients with ARDS Risk Factors

**DOI:** 10.1155/2018/1739615

**Published:** 2018-06-12

**Authors:** Zhi Xu, Guo-Ming Wu, Qi Li, Fu-Yun Ji, Zhong Shi, Hong Guo, Jin-Bo Yin, Jian Zhou, Liang Gong, Chun-Xia Mei, Guan-Song Wang

**Affiliations:** ^1^Institute of Respiratory Disease, Department of Respiratory, Respiratory Intensive Care Unit, Xinqiao Hospital, Third Military Medical University, Chongqing 400037, China; ^2^Emergency Department, Xinqiao Hospital, Third Military Medical University, Chongqing 400037, China; ^3^Gastroenterology Department, Xinqiao Hospital, Third Military Medical University, Chongqing 400037, China; ^4^Department of Neurosurgery, Neurosurgical Intensive Care Unit, Xinqiao Hospital, Third Military Medical University, Chongqing 400037, China; ^5^Intensive Care Unit, Daping Hospital, Third Military Medical University, Chongqing 400042, China; ^6^Department of Respiratory, Southwest Hospital, Third Military Medical University, Chongqing 400038, China; ^7^Department of Respiratory, Chongqing Traditional Chinese Medicine Hospital, Chongqing 400011, China

## Abstract

To investigate the predictive value of the acute physiology and chronic health evaluation 2 (APACHE2) score and lung injury prediction score (LIPS) for acute respiratory distress syndrome (ARDS) when combined with biomarkers for this condition in patients with ARDS risk factors. In total, 158 Han Chinese patients with ARDS risk factors were recruited from the Respiratory and Emergency Intensive Care Units. The LIPS, APACHE2 score, primary diagnosis at admission, and ARDS risk factors were determined within 6 h of admission, and PaO_2_/FiO_2_ was determined on the day of admission. Blood was collected within 24 h of admission for the measurement of angiopoietin-2 (ANG-2), sE-selectin, interleukin-6 (IL-6), and interleukin-8 (IL-8) levels. ARDS was monitored for the next 7 days. Univariate and multivariate analyses and receiver operating characteristic (ROC) analyses were employed to construct a model for ARDS prediction. Forty-eight patients developed ARDS within 7 days of admission. Plasma ANG-2 level, sE-selectin level, LIPS, and APACHE2 score in ARDS patients were significantly higher than those in non-ARDS patients. ANG-2 level, LIPS, and APACHE2 score were correlated with ARDS (*P* < 0.001, *P* < 0.006, and *P* < 0.042, resp.). When the APACHE2 score was used in combination with the LIPS and ANG-2 level to predict ARDS, the area under the ROC curve (AUC) was not significantly increased. Compared to LIPS or ANG-2 alone, LIPS in combination with ANG-2 had significantly increased positive predictive value (PPV) and AUC for the prediction of ARDS. In conclusion, plasma ANG-2 level, LIPS, and APACHE2 score are correlated with ARDS. Combined LIPS and ANG-2 level displays favorable sensitivity, specificity, and AUC for the prediction of ARDS.

## 1. Introduction

Acute respiratory distress syndrome (ARDS) is a critical illness characterized by noncardiogenic pulmonary edema and refractory hypoxemia [1]. Although great progress has been made in the methods used to improve the clinical prognosis of ARDS (such as the use of protective mechanical ventilation [2–4] and fluid balance therapy [5]), the morbidity and mortality of ARDS remain largely unchanged. Thus, early prediction and early therapy for ARDS will be helpful for reducing morbidity and mortality [6].

Unfortunately, although a variety of ARDS studies have been conducted, there is no favorable prediction model for ARDS. The multicenter study by Gajic et al. included more than 5000 cases, and the investigators constructed a predictor of ARDS: the lung injury prediction score (LIPS) [7, 8]. However, the positive predictive value (PPV) of the LIPS was only 0.18, thereby limiting its clinical application. Other predictors of ARDS (such as early acute lung injury (ALI) and surgical lung injury prediction models) are not validated in clinical practice [9, 10].

ARDS is an uncontrollable pulmonary inflammation characterized by neutrophil activation and endothelial injury [11–13]. Plasma interleukin-6 (IL-6) and interleukin-8 (IL-8) in ARDS patients are significantly higher than those in patients without ARDS [14–16]. Plasma angiopoietin-2 (ANG-2) is a proinflammatory cytokine that can regulate endothelial permeability [17]. Serum ANG-2 level is significantly increased in ARDS patients [18, 19], and ANG-2 displays predictive value for ARDS [19, 20]. However, numerous other factors also affect the outcomes of ARDS, and no single biomarker has been found to predict ARDS onset.

We hypothesized that the combined use of two or more parameters would be better than using only one factor in predicting ARDS. Thus, in the present study, the predictive value for ARDS by combining LIPS with one or more of 4 biomarkers was investigated.

## 2. Materials and Methods

### 2.1. Study Population

**Figure 1:**
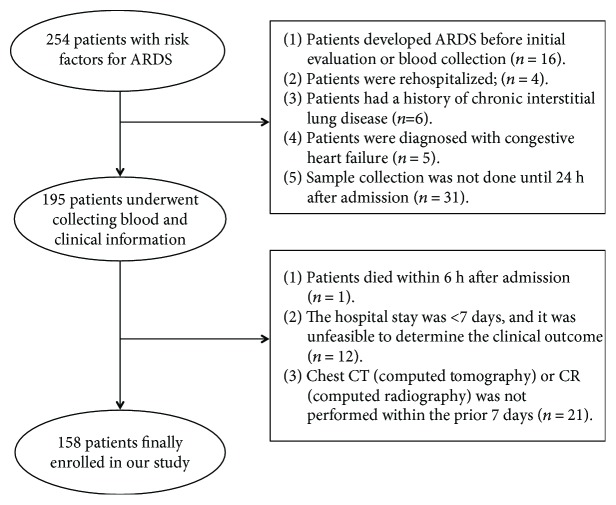
Details of subject enrollment and reason for exclusion from the present study.

In this prospective study, 254 Han Chinese patients with risk factors for ARDS were recruited from the Respiratory Intensive Care Unit (RICU) and Emergency Intensive Care Unit (EICU) of Xinqiao Hospital, Daping Hospital, and Southwest Hospital of the Third Military Medical University, between March 2013 and May 2016. The inclusion criterion was one or more risk factors for ARDS in the patients [8]. The exclusion criteria were as follows: (1) patients who developed ARDS before initial evaluation or blood collection (*n* = 16); (2) patients who were rehospitalized (*n* = 4); (3) the hospital stay was shorter than 7 days, and it was unfeasible to determine the clinical outcome (*n* = 12); (4) patients who died within 6 h of admission (*n* = 1); (5) patients had a history of chronic interstitial lung disease (*n* = 6) or were diagnosed with congestive heart failure (*n* = 5); (6) chest computed tomography (CT) or computed radiography (CR) was not performed within the prior 7 days (*n* = 21); and (7) sample collection was not performed until 24 h of admission (*n* = 31). Patients fulfilling one or more of the above conditions were excluded from the study. Finally, 158 patients were enrolled into our study (Figure 1). This study was approved by the Ethics Committee of the Third Military Medical University. Informed consent was obtained from each patient or the patient's relatives before the study.

### 2.2. Sample Collection

Blood was collected within 24 h of admission into the RICU or EICU, and plasma was separated and stored at −80°C.

### 2.3. Biomarker Measurements

Plasma concentrations of ANG-2, sE-selectin, IL-6, and IL-8 were measured by commercial ELISA kits (Cusabio, China) according to the manufacturer's instructions as follows: standards for ANG-2, sE-selectin, IL-6, and IL-8 were prepared for generating corresponding standard curves. In each well, 100 *μ*l sample or standard was added and the plate was sealed using a membrane for 90 min of reaction at 37°C. Then, 100 *μ*l biotin-labeled anti-rat antibodies was added for 60 min of reaction at 37°C. Subsequently, 300 *μ*l washing buffer was added, and after the mixture had soaked into the plate for 1 min, the buffer was discarded. In each well, 90 *μ*l color development solution was added, and the plate was sealed using a membrane and placed in the dark for 30 min of reaction at 37°C. Thereafter, 100 *μ*l termination solution was added, and the color of the solution turned from blue to yellow. Samples were read at 450 nm using a microplate reader. Values were calculated based on a standard curve constructed for each assay.

### 2.4. Clinical Data Collection

Baseline clinical information, including age, sex, admission source, primary diagnosis at admission, ARDS risk factors, ARDS risk modifiers, and other parameters, was collected within 6 h of admission into the RICU or EICU (Table 1). The LIPS was calculated within 6 h of admission as previously reported [8]. The LIPS has two indexes including 22 categories, such as shock, aspiration, and sepsis. The scores range from 0 to 15.5. The acute physiology and chronic health evaluation 2 (APACHE2) score was calculated within 24 h of admission. The APACHE2 score has three categories, namely, acute physiology score, age score, and chronic health score [21]. The scoring was performed by 2 investigators in this study who were blinded to the measurement and expression of biomarkers.

### 2.5. Primary Outcome and Definitions

The primary endpoints were ARDS onset within 7 days and clinical outcomes of ARDS within 60 days. The primary endpoints were determined by two experienced clinicians who were blinded to the expression of the plasma biomarkers. ARDS was diagnosed according to the Berlin definition for ARDS (2010) [1]. Sepsis, severe sepsis, and septic shock were diagnosed according to the criteria of the American College of Chest Physicians/Society of Critical Care Medicine Consensus Conference [22].

### 2.6. Statistical Analysis

Statistical analysis was performed with SPSS version 20.0. Continuous data were expressed as the mean ± standard deviation and categorical data as numbers. Comparisons of continuous data were performed with the *t*-test or Student's *t*-test and of categorical data with the chi-square test or Fisher's exact test between two groups (ARDS group and non-ARDS group, the group of patients with and without ARDS, resp.). Univariate and multivariate logistic regression analyses were employed to identify factors associated with ARDS. For the establishment of the model with the LIPS and ANG-2, the probability value (*P* value) was obtained from logistic regression analysis and then used as a new indicator for the diagnosis of ARDS based on receiver operating characteristic (ROC) curve analysis. The accuracy of diagnosis was determined using area under the ROC curve (AUC; 95% confidence interval (CI) and *P* value < 0.05). Linear regression analysis was used for determining correlations. A value of *P* < 0.05 was considered statistically significant.

## 3. Results

### 3.1. Clinical Information and Patient Characteristics at Baseline

#### 3.1.1. Baseline Characteristics of Patients

A total of 254 patients with risk factors of ARDS were recruited, and 158 patients were included for final analysis. The incidence of ARDS was 28.5% within 7 days of admission (45/158). As shown in Table 1, there were no significant differences in age, sex, initial diagnosis, or risk factors between ARDS and non-ARDS groups. However, the APACHE2 score, LIPS, use of invasive mechanical ventilation, and mortality within 60 days were significantly higher in the ARDS group than in the non-ARDS group, indicating that disease severity in the ARDS group is higher than that in the non-ARDS group.

#### 3.1.2. Characteristics of Patients in Different Groups at Baseline

Preexisting medical interventions and therapies before evaluation are important factors affecting the accuracy of a prediction model. However, whether patients receive prior interventions or therapies before admission is an uncontrollable factor. Thus, a good prediction model requires the inclusion of other medical confounding factors. In the present study, patients were divided into two groups as follows: (group A) patients who had received vasopressors or different kinds of respiratory support including oxygen inhalation through the nasal tubes, noninvasive mechanical ventilation, or/and invasive mechanical ventilation before admission and (group B) patients who received no prior therapy before admission. The APACHE2 score, use of invasive mechanical ventilation, and mortality within 60 days were comparable in the 2 groups, suggesting that disease severity was similar between them (Table 2).

### 3.2. Prediction and Regression Analysis of LIPS, APACHE2 Score, and ANG-2, sE-Selectin, IL-6, and IL-8 Levels for ARDS

#### 3.2.1. LIPS, APACHE2 Score, and ANG-2, sE-Selectin, IL-6, and IL-8 Concentration in the ARDS and Non-ARDS Groups

Plasma ANG-2 level, sE-selectin level, LIPS, and APACHE2 score in the ARDS group were significantly higher than those in the non-ARDS group, but plasma IL-8 and IL-6 level was not different between the two groups (Tables 3).

#### 3.2.2. Univariate and Multivariate Regression Analyses of LIPS and Biomarkers for the Prediction of ARDS

Univariate analysis showed that ANG-2 level, sE-selectin level, APACHE2 score, LIPS, and septic shock were closely associated with ARDS (Table 4, univariate analysis). However, multivariable logistic regression analysis indicated that only ANG-2 level, LIPS, and APACHE2 score were correlated with ARDS (Table 4, multivariate regression analysis).

#### 3.2.3. Prediction of ARDS with APACHE2 Score Alone or in Combination with LIPS or ANG-2 Level

When the APACHE2 score, LIPS, and ANG-2 level were independently used to predict ARDS, the APACHE2 score had the lowest AUC (0.649). When the APACHE2 score was used in combination with LIPS or ANG-2 level for the prediction of ARDS, the AUC did not significantly increase (Tables 5 and 6). The APACHE2 score had a low AUC for the prediction of ARDS, and the APACHE2 score in combination with LIPS or ANG-2 level also failed to increase the AUC for the prediction of ARDS. Thus, the APACHE2 score was not included as a factor for the prediction of ARDS.

### 3.3. Prediction of ARDS with LIPS, ANG-2, and LIPS + ANG-2 Models

In subsequent experiments, we used LIPS, ANG-2 level, and LIPS + ANG-2 level to establish models for the prediction of ARDS. In the LIPS + ANG-2 model, the probability of LIPS and ANG-2 was obtained from logistic regression analysis (*Y* = −3.586 + 0.317∗LIPS + 0.232∗ANG-2) and then used to predict ARDS.

**Figure 2:**
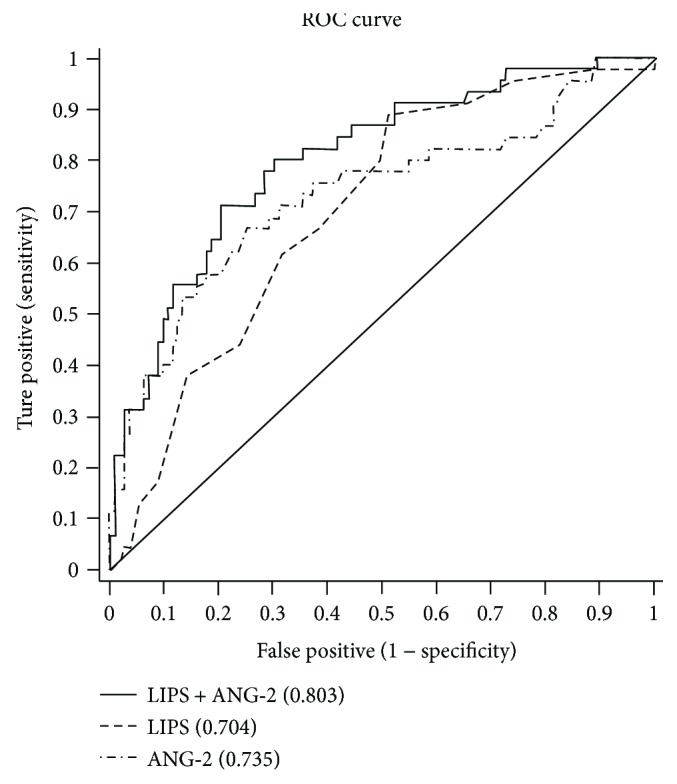
ROC of ANG-2, LIPS, and LIPS + ANG-2 for predicting ARDS. The figure depicts that the AUC for the LIPS + ANG-2 model was significantly higher than that for the LIPS or ANG-2 model, indicating that the LIPS + ANG-2 model has a better predictive value for ARDS that the LIPS and ANG-2 models.

When the cutoff value of ANG-2 level was 4.121 ng/ml, the sensitivity, specificity, and AUC were 66.67%, 75.22%, and 0.735, respectively, in predicting ARDS. The predictive value of the ANG-2 model was slightly better than that of the LIPS model. The sensitivity, specificity, and AUC were 71.11%, 79.65%, and 0.803, respectively, in predicting ARDS with the LIPS + ANG-2 with a cutoff of 0.2821. The PPV and AUC for the LIPS + ANG-2 model were significantly higher than those for the LIPS or ANG-2 model, indicating that the LIPS in combination with ANG-2 level has a better capability to predict ARDS than when either of the parameters is used alone (Table 7 and Figure 2).

### 3.4. Subgroup Analysis of the LIPS, ANG-2, and LIPS + ANG-2 Models

**Figure 3:**
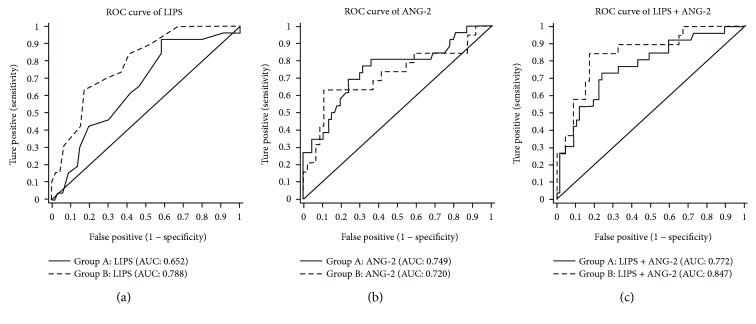
ROC curves of the LIPS, ANG-2, and LIPS + ANG-2 models for predicting ARDS in group A (solid line) and group B (dotted line). (a) AUC for the LIPS model in group A was smaller than that in group B (0.652 versus 0.788); (b) AUC for the ANG-2 model in group A was larger than that in group B (0.749 versus 0.720); (c) AUC for the LIPS + ANG-2 model in group A was smaller than that in Group B (0.772 versus 0.847).

The major difference between group A and group B was the use of medical intervention or therapy before evaluation of the LIPS or measurement of biomarkers. However, prior medical interventions or therapies may affect the accuracy of prediction models. To evaluate the influence of medical intervention or therapy on the accuracy of the above models, we performed subgroup analysis. The results showed that the LIPS + ANG-2 model had the largest AUC (0.772), and the LIPS model had the smallest AUC (0.652) in group A; the LIPS + ANG-2 model had the largest AUC (0.847), and the ANG-2 model had the smallest AUC (0.720) in group B. These results suggest that the LIPS + ANG-2 model has a better predictive value for ARDS than the LIPS or ANG-2 model regardless of prior medical intervention or therapy. The AUCs for the LIPS + ANG-2 model and the LIPS model in group A were smaller than those in group B (0.772 versus 0.847 and 0.652 versus 0.788, resp.), but the AUC for the ANG-2 model in group A was larger than that in group B (0.749 versus 0.720). These findings indicate that although the prediction of ARDS with the LIPS + ANG-2 model is affected by prior medical intervention; the LIPS + ANG-2 model has a better predictive capability for ARDS. Moreover, the prediction of ARDS with the LIPS model is also influenced by prior medical intervention. However, the prediction with the ANG-2 model does not seem to be affected by prior medical intervention, and its AUC is higher in group A (Table 8 and Figure 3).

### 3.5. Correlation of LIPS, ANG-2, and LIPS + ANG-2 Models with PaO_2_/FiO_2_

The correlation of the three prediction models with PaO_2_/FiO_2_ on the day of admission was further evaluated. Simple and binary linear regression analyses (Table 9) showed that the three models were positively correlated with severity of lung injury and that the LIPS + ANG-2 model displayed the best correlation (LIPS: *r* = −0.394, *P* < 0.001; ANG-2: *r* = −0.189, *P* = 0.018; LIPS + ANG-2: *r* = −0.426, *P* < 0.001).

## 4. Discussion

Our results showed that the LIPS, evaluated based on clinical information, could predict the occurrence of ARDS (AUC: 0.704, 95% CI: 0.618~0.789, *P* < 0.001). In addition, of the 4 investigated biomarkers of ARDS, only ANG-2 level displayed predictive value for ARDS (AUC: 0.735, 95% CI: 0.641~0.829, *P* < 0.001). The combined use of the LIPS and ANG-2 level increased the accuracy of prediction of ARDS (AUC: 0.803, 95% CI: 0.727~0.879, *P* < 0.001), and the PPV of the LIPS + ANG-2 model increased to 58.19%.

The LIPS model was proposed in 2011 by Gajic and Trillo-Alvarez for the prediction of ALI/ARDS according to their multicenter study on a large sample. It has a good predictive value for ALI (AUC: 0.80~0.84). Our results showed that the LIPS was also correlated with ARDS (odds ratio (OR): 1.324, 95% CI: 1.083~1.618, *P* = 0.006).

For patients with critical illness in the ICU, the APACHE2 score is a good parameter that can be used to predict mortality [23]. However, no study has been conducted on the usefulness of the APACHE2 score in the prediction of ARDS. In our study, the APACHE2 score was closely correlated with ARDS (OR: 1.070, 95% CI: 1.003~1.141, *P* < 0.042). However, compared with the LIPS and ANG-2 level, APACHE2 score displayed the smallest AUC for ARDS prediction. Moreover, when combined with the LIPS and ANG-2 level, APACHE2 score failed to increase the predictive power of these two parameters. Therefore, the APACHE2 score was not included for further analysis, but the LIPS was preserved.

In this study, 4 biomarkers, namely, ANG-2, sE-selectin, IL-8, and IL-6, related to the pathogenesis of ARDS were measured in blood.

ANG-2 is a secreted endothelial cell-specific growth factor. It can improve the sensitivity of vascular endothelial cells to vascular endothelial growth factors (VEGFs) and enhance angiogenesis in the presence of VEGF. On the other hand, ANG-2 can cause endothelial apoptosis, leading to vascular degeneration. Therefore, ANG-2 is an important biomarker of endothelial activation/dysfunction [24]. ANG-2 demonstrated proinflammatory activity and can regulate endothelial permeability [17]. ARDS is an uncontrollable pulmonary inflammation characterized by neutrophil activation and endothelial injury [11–13]. Increased vascular permeability and pulmonary vascular leakage are extremely important pathophysiological indicators of ARDS. Studies have shown that ANG-2 level is significantly increased in ARDS patients [18, 19]. In patients with severe sepsis, ANG-2 level is correlated with the clinical outcomes of ARDS at 28 days and can be used to predict the prognosis of ARDS [25].

IL-8 and IL-6 are important proinflammatory cytokines involved in the pathogenesis of ARDS [7, 14, 16, 26]. sE-selectin is a proinflammatory cytokine expressed on endothelial cells and is can mediate adhesion and aggregation between white blood cells and endothelial cells [27]. sE-selectin may predict ARDS with a PPV of 68% and negative predictive value (NPV) of 86% [28]. The activation and migration of neutrophils are important for the pathogenesis of ARDS.

We found that plasma ANG-2 and sE-selectin levels in ARDS patients were dramatically higher than those in non-ARDS patients, but IL-8 or IL-6 level displayed no difference between the ARDS and non-ARDS groups. Further multivariate analysis showed that only ANG-2 had a close correlation with ARDS (OR: 1.258, 95% CI:1.137~1.392, *P* < 0.001). Thus, sE-selectin, IL-8, and IL-6 were not included in the model for the prediction of ARDS, and ANG-2 was employed to establish this model.

Although our findings showed that the LIPS had the predictive capability for ARDS, its AUC was significantly lower than that reported by Gajic et al. and Trillo-Alvarez et al. [7, 8]. This difference may be explained by the fact that some patients in the present study were transferred from other hospitals, and medical intervention before the evaluation of the LIPS may have biased the results. Nevertheless, the LIPS still had a high predictive value with an AUC of 0.704.

Our results also revealed that ANG-2 level alone had favorable predictive capability for ARDS (AUC: 0.735). However, we attempted to identify a model with better predictive capability than LIPS or ANG-2 alone. Thus, we performed logistic regression analysis of the LIPS and ANG-2, and the probability value (*Y* = −3.586 + 0.317∗LIPS + 0.232∗ ANG-2) was obtained and used to predict ARDS. The results showed that, with the cutoff value of this probability of 0.2821, the AUC for the LIPS + ANG-2 model was 0.803 in predicting ARDS, which was higher than that for the LIPS or ANG-2 model alone. In addition, the LIPS + ANG-2 model had higher PPV and NPV than did the LIPS or ANG-2 model.

Unlike the study by Agrawal et al. [29], we further investigated whether the LIPS in combination with ANG-2 level had different predictive capabilities in patients with or without medical intervention before admission. The results showed that the prediction of ARDS with the LIPS model but not with the ANG-2 model was affected by prior medical intervention. Moreover, the predictive capability of the LIPS + ANG-2 model for ARDS was better than that of the LIPS or ANG-2 model alone, regardless of prior medical intervention. However, prior medical interventions actually affected the accuracy of the LIPS + ANG-2 model in the prediction of ARDS, and its AUC was reduced by 7%. Nevertheless, the LIPS + ANG-2 model had a good predictive capability for ARDS in group A. Thus, we speculate that the LIPS + ANG-2 model would be more suitable for predicting ARDS in complex clinical situations.

Although strict inclusion and exclusion criteria were used in the present study to establish a better prediction model than the LIPS or ANG-2 model, our study had several limitations. (1) The volume of blood collected was relatively small, and thus it was impossible to detect all biomarkers for ARDS (such as biomarkers related to epithelial injury, endothelial injury, and other inflammatory factors). (2) The time and location of sample collection were limited, and we failed to dynamically observe changes in plasma biomarkers or compare plasma biomarkers with bronchoalveolar lavage fluid (BALF) biomarkers, which may have limited our understanding of these biomarkers. (3) The sample size was small. We need to expand the sample size in future studies. Nevertheless, our study had some advantages. This was a multicenter study in which patients were recruited from three general hospitals. In addition, the exclusion criteria were strict and excluded most clinical confounding factors to make our results reliable. Furthermore, patients who received medical intervention before admission were also recruited in the present study. These patients are special but are common in ICUs. Thus, our results are more likely to be widely applicable.

## 5. Conclusions

Taken together, our results demonstrate that the combined use of a clinical scoring system and biomarkers of ARDS is helpful for the early prediction of ARDS and might become a future research direction in the establishment of models for ARDS prediction. Compared with the LIPS model and the ANG-2 model, the LIPS + ANG-2 model had the best predictive capability for ARDS. In subgroup analysis, the results revealed that the ANG-2 model and the LIPS + ANG-2 model were applicable in patients with medical intervention before admission and that the LIPS + ANG-2 model had better predictive capability than the ANG-2 model. Our findings are helpful for the early identification of patients at high risk for ARDS and early prevention and management of ARDS.

## Figures and Tables

**Table 1:** Baseline characteristics of patients in the ARDS and non-ARDS groups.

Variable	Non-ARDS group (*n* = 113)	ARDS group (*n* = 45)	*P* value
Age, yr	58.5 ± 20.3	60.0 ± 17.1	0.107
Male	82 (72.6%)	35 (77.8%)	0.500
Ethnicity	Han (100.0%)	Han (100.0%)	1.000
Patients resource			0.979
Family	46 (40.7%)	19 (42.2%)	0.861
Other departments	18 (15.9%)	7 (15.6%)	0.954
General wards of the respiratory department	2 (1.8%)	1 (2.2%)	0.851
Other hospital	47 (41.6%)	18 (40.0%)	0.854
Primary diagnosis at admission			0.364
Respiratory	59 (52.2%)	31 (68.9%)	0.056
Trauma	31 (27.4%)	6 (13.3%)	0.059
Other	7 (6.2%)	3 (6.7%)	0.912
Acute abdominal disease	7 (6.2%)	1 (2.2%)	0.304
Cardiopulmonary resuscitation	3 (2.7%)	1 (2.2%)	0.878
Operation	6 (5.3%)	3 (6.7%)	0.740
Predisposing conditions			
Category			0.128
Shock	7 (6.2%)	5 (11.1%)	0.292
Sepsis	34 (30.1%)	23 (51.1%)	0.013^∗^
Pancreatitis	5 (4.4%)	1 (2.2%)	0.513
Pneumonia	82(72.6%)	38 (84.4%)	0.115
High-risk surgery	3 (2.7%)	1 (2.2%)	0.876
Trauma	31 (27.4%)	5 (11.1%)	0.027^∗^
Number			0.580
Include 1 factor: *n* (%)	57 (50.4%)	19 (40.2%)	0.351
Include 2 factors: *n* (%)	43 (38.1%)	20 (44.4%)	0.459
Include 3 factors: *n* (%)	11 (9.7%)	6 (13.3%)	0.510
Include 4 factors: *n* (%)	2 (1.8%)	0 (0.0%)	0.369
APACHE2 score	14.7 ± 6.0	18.5 ± 7.2	0.001^∗∗^
LIPS	4.4 ± 2.1	5.6 ± 1.8	0.001^∗∗^
60-day outcome	16 (14.2%)	21 (46.7%)	<0.001^∗∗^
Use of vasopressors	23 (20.3%)	19 (42.2%)	0.005^∗∗^
Methods of respiratory support			<0.001^∗∗^
Oxygen inhalation through the nasal tube	33 (29.2%)	14 (31.1%)	0.813
Noninvasive ventilation	25 (22.1%)	10 (22.2%)	0.989
Invasive mechanical ventilation	48 (42.5%)	21 (46.7%)	0.632
Noninvasive and invasive mechanical ventilation	7 (6.2%)	13 (28.9%)	<0.001^∗∗^

^∗^ *P* < 0.05, ^∗∗^*P* < 0.01.

**Table 2:** Baseline characteristics of patients who received prior therapy and those who did not.

Variable	Group A (*n* = 93)	Group B (*n* = 65)	*P* value
Age, yr	62.9 ± 18.1	56.0 ± 20.8	0.028^∗^
Male	72 (77.4%)	45 (69.2%)	0.248
Primary diagnosis at admission			0.018^∗^
Respiratory	56 (60.2%)	34 (52.3%)	0.323
Trauma	17 (18.3%)	20 (30.8%)	0.068
Acute abdominal disease	2 (2.2%)	6 (9.2%)	0.046^∗^
Cardiopulmonary resuscitation	2 (2.2%)	2 (3.1%)	0.715
Operation	9 (9.7%)	0 (0.0%)	0.011^∗^
Other	7 (7.5%)	3 (4.6%)	0.460
Predisposing conditions			
Category			0.088
Shock	8 (8.6%)	4 (6.2%)	0.568
Sepsis	40 (43.0%)	17 (26.2%)	0.030^∗^
Pancreatitis	2 (2.2%)	4 (6.2%)	0.195
Pneumonia	79 (84.9%)	41 (63.1%)	0.002^∗∗^
High-risk surgery	3 (3.2%)	1 (1.5%)	0.506
Trauma	16 (17.2%)	20 (30.8%)	0.046^∗^
Number			0.563
Include 1 factor: *n* (%)	42 (45.2%)	34 (52.3%)	0.376
Include 2 factors: *n* (%)	39 (41.9%)	24 (36.9%)	0.527
Include 3 factors: *n* (%)	10 (10.8%)	7 (10.8%)	0.997
Include 4 factors: *n* (%)	2 (2.2%)	0 (0.0%)	0.234
APACHE2 score	15.9 ± 6.6	15.6 ± 6.6	0.743
LIPS	5.1 ± 2.2	4.2 ± 1.8	0.017^∗^
60-day outcome	23 (24.7%)	14 (21.5%)	0.641
Morbidity of ARDS	26 (28.0%)	19 (29.2%)	0.861

Group A: patients who had received therapy before admission; group B: patients who had not received therapy before admission. ^∗^*P* < 0.05, ^∗∗^*P* < 0.01.

**Table 3:** LIPS, APACHE2 score, and levels of ANG-2, sE-selectin, IL-6, and IL-8 in the ARDS and non-ARDS groups.

	ARDS (*n* = 45)	Non-ARDS (*n* = 113)	*t*-test or Student's *t*-test	*P* value
ANG-2 (ng/ml)	7.36 ± 5.99	3.05 ± 2.98	4.601	<0.001
IL-8 (pg/ml)	97.82 ± 188.99	73.16 ± 314.70	0.491	0.624
IL-6 (pg/ml)	117.54 ± 182.08	79.32 ± 132.47	1.464	0.145
sE-selectin (ng/ml)	10.61 ± 5.56	7.84 ± 5.18	2.968	0.003
APACHE2 score	18.5 ± 7.2	14.7 ± 6.0		0.001
LIPS	5.6 ± 1.8	4.4 ± 2.1		0.001

ANG-2: angiopoietin-2; IL: interleukin; LIPS: lung injury prediction score. Data are presented as the mean ± SD or *n* (%). Analysis performed using *t*-test or Student's *t*-test.

**Table 4:** Univariate and multivariate regression analyses of LIPS and prediction of ARDS.

	Univariate regression analyses				Multivariate regression analyses			
	OR	95% CI of OR	*χ* ^2^	*P* value	OR	95% CI of OR	*χ* ^2^	*P* value
ANG-2 (ng/ml)	1.252	1.138~1.377	21.289	<0.001	1.258	1.137~1.392	19.702	<0.001
IL-8 (pg/ml)	1.000	0.999~1.001	0.234	0.628				
IL-6 (pg/ml)	1.002	0.999~1.004	2.017	0.156				
sE-selectin (ng/ml)	1.097	1.028~1.170	7.866	0.005				
APACHE2 score	1.092	1.034~1.154	10.004	0.002	1.070	1.003~1.141	4.150	0.042
LIPS	1.344	1.123~1.610	10.338	0.001	1.324	1.083~1.618	7.520	0.006
Sepsis	1.141	0.457~2.850	0.080	0.777				
Severe sepsis	1.444	0.456~4.573	0.391	0.532				
Sepsis shock	4.327	1.531~12.225	7.639	0.006				
Infection-related ARDS risk	2.343	0.994~5.527	3.783	0.052				
Invasive mechanical ventilation	1.575	0.786~3.157	1.639	0.200				

**Table 5:** Prediction of ARDS with the APACHE2 score alone or in combination with LIPS or ANG-2.

	Cutoff	TPR	TNR	PV+	PV−	AUC	SE	95% CI	*P* value
APACHE2	16.5000	0.5333	0.6460	0.3750	0.7766	0.649	0.048	0.555~0.743	0.003
ANG-2	4.1210	0.6667	0.7522	0.5172	0.8500	0.735	0.048	0.641~0.829	<0.001
LIPS	5.2500	0.6222	0.6814	0.4375	0.8191	0.704	0.044	0.618~0.789	<0.001
ANG-2 + APACHE2	0.2887	0.7111	0.7788	0.5614	0.8713	0.795	0.038	0.721~0.869	<0.001
LIPS + APACHE2	0.2409	0.7556	0.5664	0	0.8534	0.707	0.044	0.622~0.793	<0.001

**Table 6:** AUC for the APACHE2 score alone or in combination with LIPS or ANG-2 level in predicting ARDS.

	*Z*	*P* value
ANG-2 versus ANG-2 + APACHE2	0.9801	0.3271
LIPS versus LIPS + APACHE2	0.0482	0.9615

**Table 7:** Characteristics of ANG-2, LIPS, and LIPS + ANG-2 models for predicting ARDS.

	Cutoff	TPR	TNR	PPV	NPV	AUC	SE	95% CI	*P* value
LIPS	5.2500	0.6222	0.6814	0.4375	0.8191	0.704	0.044	0.618~0.789	<0.001
ANG-2	4.1210	0.6667	0.7522	0.5172	0.8500	0.735	0.048	0.641~0.829	<0.001
LIPS + ANG-2	0.2821 (*Y*)	0.7111	0.7965	0.5819	0.8738	0.803	0.039	0.727~0.879	<0.001

*Y* = −3.586 + 0.317∗LIPS + 0.232∗ANG-2.

**Table 8:** Subgroup analysis for the prediction of ARDS with the LIPS, ANG-2, and LIPS + ANG-2 models.

		Group A			Group B	
	LIPS	ANG-2	LIPS + ANG-2	LIPS	ANG-2	LIPS + ANG-2
Cutoff	5.2500	3.1110	0.2827	5.2500	5.9235	0.2392
TPR	0.6154	0.7692	0.7308	0.6316	0.6316	0.8421
TNR	0.5821	0.6866	0.7612	0.8261	0.8913	0.8261
PPV	0.3697	0.4943	0.5493	0.5912	0.6982	0.6585
NPV	0.7917	0.8819	0.8766	0.8492	0.8587	0.9293
AUC	0.652	0.749	0.772	0.788	0.720	0.847
95% CI	0.532~0.772	0.631~0.868	0.664~0.881	0.675~0.902	0.566~0.873	0.742~0.952
*P* value	0.023	<0.001	<0.001	<0.001	0.006	<0.001

Group A: patients who had received prior therapy before admission; group B: patients who had not received prior therapy before admission.

**Table 9:** Correlation of the LIPS, ANG-2, and LIPS + ANG-2 models with PaO_2_/FiO_2_.

	LIPS	ANG-2	LIPS + ANG-2
Correlation coefficient	−0.394	−0.189	−0.426
*P* value	<0.001	0.018	<0.001

## Abbreviations

ANG-2: Angiopoietin-2
IL: Interleukin
LIPS: Lung injury prediction score
ARDS: Acute respiratory distress syndrome
APACHE2: Acute physiology and chronic health evaluation 2
PPV: Positive predictive values
AUC: Area under the receiver operating characteristic
ROC: Receiver operating characteristic.
